# Teeth and prostheses detection in dental panoramic X-rays using CNN-based object detector and a priori knowledge-based algorithm

**DOI:** 10.1038/s41598-023-43591-z

**Published:** 2023-10-02

**Authors:** Md. Anas Ali, Daisuke Fujita, Syoji Kobashi

**Affiliations:** Graduate School of Engineering, University of Hyogo, Himeji, Japan

**Keywords:** Engineering, Mathematics and computing

## Abstract

Deep learning techniques for automatically detecting teeth in dental X-rays have gained popularity, providing valuable assistance to healthcare professionals. However, teeth detection in X-ray images is often hindered by alterations in tooth appearance caused by dental prostheses. To address this challenge, our paper proposes a novel method for teeth detection and numbering in dental panoramic X-rays, leveraging two separate CNN-based object detectors, namely YOLOv7, for detecting teeth and prostheses, alongside an optimization algorithm to refine the outcomes. The study utilizes a dataset of 3138 radiographs, of which 2553 images contain prostheses, to build a robust model. The tooth and prosthesis detection algorithms perform excellently, achieving mean average precisions of 0.982 and 0.983, respectively. Additionally, the trained tooth detection model is verified using an external dataset, and six-fold cross-validation is conducted to demonstrate the proposed method’s feasibility and robustness. Moreover, the investigation of performance improvement resulting from the inclusion of prosthesis information in the teeth detection process reveals a marginal increase in the average F1-score, rising from 0.985 to 0.987 compared to the sole teeth detection method. The proposed method is unique in its approach to numbering teeth as it incorporates prosthesis information and considers complete restorations such as dental implants and dentures of fixed bridges during the teeth enumeration process, which follows the universal tooth numbering system. These advancements hold promise for automating dental charting processes.

## Introduction

A dental panoramic X-ray provides a wide-angle view of the oral cavity, including the teeth, jaws, temporomandibular joints, and surrounding tissues. Dentists use dental panoramic radiographs in various aspects of dental care, such as planning oral surgeries, diagnosing periodontal disease, evaluating heavily restored dentition, assessing facial trauma, and conducting initial patient assessments^1^. However, interpreting and diagnosing severe dental conditions from panoramic X-rays is challenging due to complex anatomical structures, pathologies, and imaging distortions^2^. Moreover, the manual process of recording diagnostic information for each tooth in a paper chart is time-consuming, tedious, and prone to human error. Therefore, tooth detection and automatic diagnosis are highly desirable to improve dental health care quality^3^. Additionally, automatic tooth detection and diagnosis can aid dentists in maintaining electronic records of oral health over time and identifying potential issues before they worsen. Furthermore, digital teeth profiling can also be utilized for personal identification in preparation for large-scale disasters or other oral forensic purposes^4,5^.

In recent years, a specialized area of artificial intelligence known as deep learning has been intensively applied in many healthcare fields with the aim of automation^6–10^. Like other clinical fields, the application of deep learning in dentistry, such as diagnosing caries, fractures, periodontal bone loss, and periapical lesions in dental images, is rapidly growing^11–16^. In addition, it has emerged as a promising tool for teeth detection in dental X-ray images^17^. Deep learning-based teeth detection has several benefits, such as being capable of training on large annotated panoramic X-ray images to learn robust representations of dental anatomy. This allows for the accurate detection of teeth despite variations in imaging conditions such as radiation exposure, resolution, and patient positioning. In a study, Chen et al.^18^ employed faster R-CNN^19^ with Inception Resnet version-2^20^ for detecting teeth and assigning them numbers according to Fédération Dentaire Internationale notation (FDI)^21^ in dental periapical film. They incorporated various post-processing techniques, such as a filtering algorithm to remove overlapping detections associated with the same tooth, a neural network model to detect missing teeth, and a rule-based module to refine the extracted results to improve the baseline detection performance. The study found a high precision rate of 0.988 for teeth detection but a relatively lower precision rate of 0.917 for numbering. Bilgir et al.^22^ also utilized the same deep learning architecture for teeth detection and enumeration in 2,482 panoramic dental X-rays. The reported precision and recall values were about 95%. Additionally, Tuzoff et al.^23^ used a faster R-CNN for teeth detection and a convolutional neural network VGG-16^24^ for numbering according to FDI notation in 1,574 cases. To improve the outcome, they applied a heuristic-based algorithm. Although the method succeeded in teeth detection and classification with sensitivity values of 0.994 and 0.98, respectively, it could not detect restorations, such as dental implants and dentures of fixed bridges. The researchers^25^ applied a single-shot detector (SSD)^26^ with an attention branch for automatic teeth detection and classification in 950 dental panoramic radiographs. Afterwards, they performed post-processing to merge the outcomes of both networks and classified them into 32 categories. However, the detection and numbering process did not consider the implants and dentures. In another study, Chung et al.^27^ used 818 cases to recognize 32 tooth types by predicting their positions regardless of missing teeth. Although the recall of 97.2% for teeth recognition was reported, they did not distinguish between boxes with teeth present and absent. The limitations of previous research, particularly the exclusion of dental implants and dentures of fixed bridges from enumeration, posed challenges for dentists in accurately determining the true number of missing teeth. Although Chen et al. in the study^28^ developed an advanced image cropping technique combined in conjunction with CNN models (AlexNet^29^, GoogLeNet^30^, and SqueezeNe^31^) to classify missing, treated, and normal teeth from cropped images, they did not incorporate teeth numbering. Other studies have solely focused on classifying disease^32^ and restoration types^33^ without teeth numbering. However, DENTECT^34^ and the model proposed by Muresan et al.^35^ could detect and enumerate teeth as well as dental therapies in panoramic X-ray images. Still, their performance in detecting and numbering dentition was poor. Moreover, they utilized limited data for teeth detection and classification purposes. Furthermore, dental prostheses distort regular teeth shapes in radiographs, making accurate detection and classification difficult, as reported by Mahdi et al.^36^.

The current research overcomes the constraints of prior studies by implementing an advanced object detection algorithm known as YOLOv7^37^ in combination with a robust optimization algorithm for teeth detection purposes. However, the novelty of our research lies in incorporating dental prosthesis information into the teeth detection process for radiographs featuring prostheses, achieved through the integration of another dedicated YOLOv7-based prosthesis detector. The central objective of this integration has two main aspects: enhancing teeth detection performance by revisiting the detection of treated teeth and facilitating teeth numbering, even when dealing with complete restorations such as implants and dentures of the fixed bridges. Incorporating complete restorations in the enumeration process provides dentists with more precise and reliable information, including the actual count of missing teeth. To achieve these objectives, a large dataset comprising 3138 panoramic images, of which 2553 contained prostheses, is leveraged to construct a resilient teeth detection and enumeration model. This novel approach holds promise for the automation of dental charting procedures.

The subsequent sections of the manuscript are arranged in the following manner: The “Materials” section introduces the universal tooth numbering system and the dataset, providing a detailed explanation of data segmentation and the annotation process. The subsequent “Method” section elaborates on the proposed teeth detection and enumeration approach in conjunction with two CNN-based object detectors. It also outlines the optimization algorithm employed in this study and the metrics used to evaluate the proposed method’s fitness. The “Results” section presents the findings from two distinct experimental analyses. In the ensuing “Discussions” section, the simulation outcomes derived from these results are elaborated upon. Finally, the paper concludes in the “Conclusion” section with summaries, limitations, and future research suggestions.

## Materials

**Figure 1:**
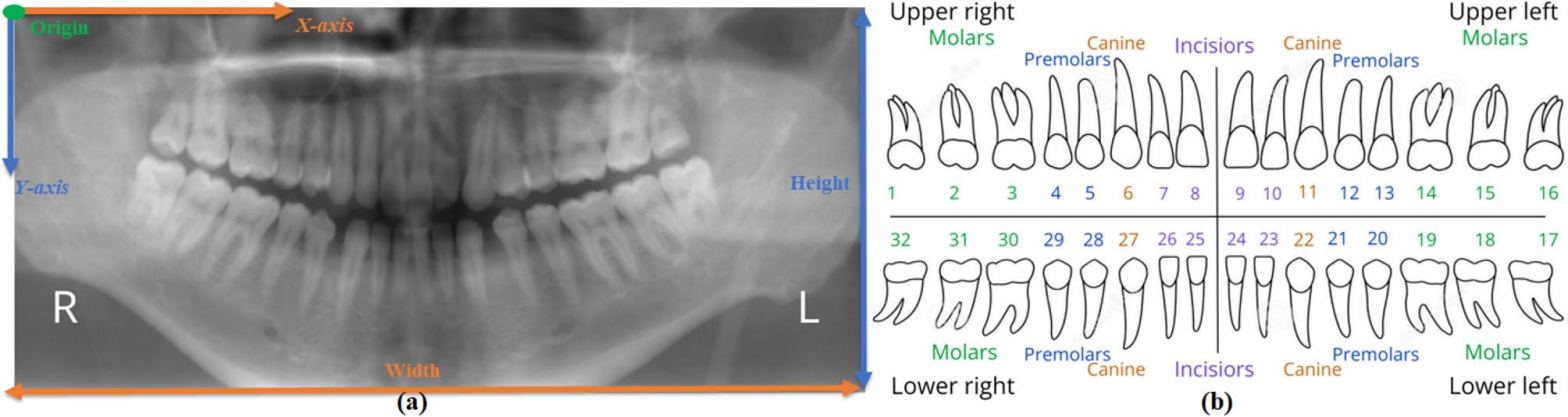
(**a**) Dental panoramic X-ray image and (**b**) Universal tooth numbering system.

This study employed dental panoramic radiographs for teeth detection, leveraging their unique capability to capture all the teeth within a single frame. As depicted in Fig. 1a, a typical dental panoramic X-ray image shows reversed left and right sides. The figure further introduces a coordinate system, with the upper left corner serving as the origin, the horizontal axis denoted as *X*, and the vertical axis as *Y*. To systematically categorize all 32 teeth, the researcher adopted the universal tooth numbering (UTN) system^38^. In this classification scheme, the upper right third molar through the upper left third molar corresponds to teeth numbered 1 through 16, while the lower left third molar through the lower right third molar corresponds to teeth numbered 17 through 32. For visual clarity, Fig. 1b illustrates the UTN system. The following section provides a concise overview of the dataset, details of data splitting, and the annotation process.

### Dataset description

**Figure 2:**
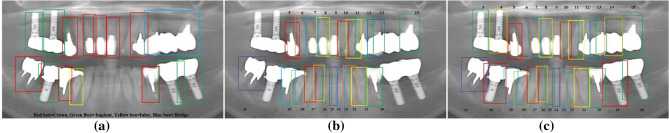
(**a**) Prosthesis annotation [Red: Crown, Green: Implant, Yellow: Inlay, and Blue: Bridge] (**b**) Tooth annotation and (**c**) All annotation ground truths.

The research commenced by collecting 3818 anonymous dental panoramic X-ray images from various dental clinics, following ethical guidelines and obtaining proper permissions. These X-ray images displayed a variety of dental conditions, including healthy teeth, supernumerary teeth, damaged/broken teeth, residual roots, and the presence of braces and dental prostheses. However, this study mainly concentrated on four types of dental prostheses: inlays (including basic fillings and onlays), crowns, implants, and bridges. Inlays fill larger cavities than the basic fillings, while onlays or partial crowns cover one or more tooth cusps. Dental crowns are tooth-shaped caps used to restore severely damaged teeth, and both inlays and crowns are partial restorations aiding in decay prevention. In contrast, dental bridges and implants^39^ are the complete restorations for replacing missing teeth. Dental bridges are false teeth held by abutment teeth on either side of a gap, and implants are artificial teeth roots inserted into the jawbone. Notably, prosthetic materials like metal and gold, being harder than natural teeth, can appear whitish in radiographs, causing distortion in teeth shape and treatment scars. An example of the four types of dental prostheses considered in this study is demonstrated in Fig. 2a.

**Figure 3:**
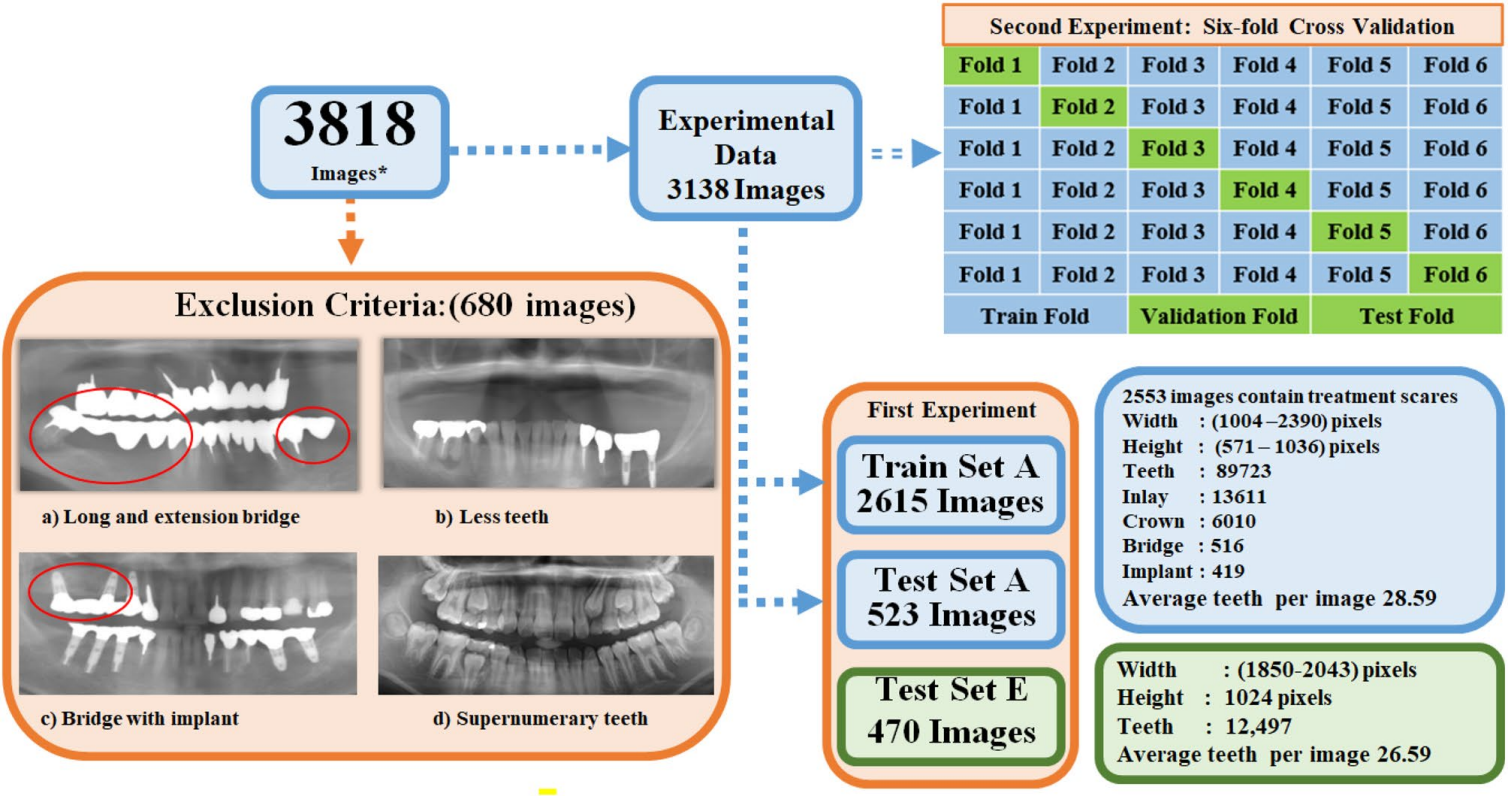
Data splitting and selection criteria.

Data selection involved images capturing the lower face edge and eyeball in a front-facing view, including those containing a bridge compensating for a single missing tooth. Images with a minimum of five upper and lower teeth were exclusively chosen to facilitate better tracing of the occlusal curve, a process elucidated in the method section. Exclusions were made for subjects with deciduous or supernumerary teeth, and images exhibiting blurriness, superimpositions, distortions, or technician-related issues. As a result, a dataset of 3138 images was curated from the initial 3818 panoramic radiographs, with 2553 displaying treatment scars. Additionally, a separate dataset denoted as test set E^40^ was established to assess our dataset’s universality and evaluate the tooth candidate detection model’s performance, as detailed in the method section. Set E comprised 598 panoramic radiographs in JPEG format, of which 470 images were annotated for evaluation, resulting in a total of 12,497 tooth instances. The remaining images were excluded due to insufficient teeth or the presence of supernumerary teeth. Additional details regarding the data description are available in Fig. 3.

### Data splitting

In order to validate the research objective, two distinct experiments were carried out. In the first experiment, the 3138 selected X-ray images were divided into train set A and test set A, consisting of 2615 and 523 images, respectively. Train set A was utilized for training the tooth and prosthesis candidate detection models, while test sets A and E were employed for evaluation. In the second experiment, the 3138 radiographs were randomly divided into six folds to evaluate the performance of the proposed method and investigate the performance improvement resulting from the addition of prosthesis information in the teeth detection process through a six-fold cross-validation experiment. This partitioning involved five folds in the training dataset and one-fold in the test dataset. The candidate detection models were trained on the training dataset, and their performance was subsequently analyzed and validated on the test dataset. Figure 3 exhibits a block diagram that depicts the data-splitting process.

### Ground truth preparation

At the beginning of the data annotation process, two sets of ground truths, “Tooth Annotation” and “Prosthesis Annotation”, were meticulously prepared using selected X-ray images through manual efforts. Within this meticulous process, each tooth was discreetly identified by drawing a rectangular bounding box around it and ascribed a class according to the UTN system under the expert guidance of a medical professional. The “Tooth Annotation” process deliberately omitted severely broken or damaged teeth, residual roots, and comprehensive restorations like implants and dentures of fixed bridges. In contrast, the “Prosthesis Annotation” focused solely on labeling four types of prostheses: inlay, crown, implant, and bridge, with bridges encompassing supportive teeth and denture portions. These ground truths served as the training and testing data for the tooth and prosthesis candidate detection models, respectively. A third ground truth, “All Annotation”, was subsequently generated to evaluate the proposed model. In this dataset, complete restorations such as implants and dentures of fixed bridges were incorporated into the “Tooth Annotation” file, categorizing them based on their positions within the jawbones. A visual representation of the data annotation process is referred to in Fig. 2.

## Method

**Figure 4:**
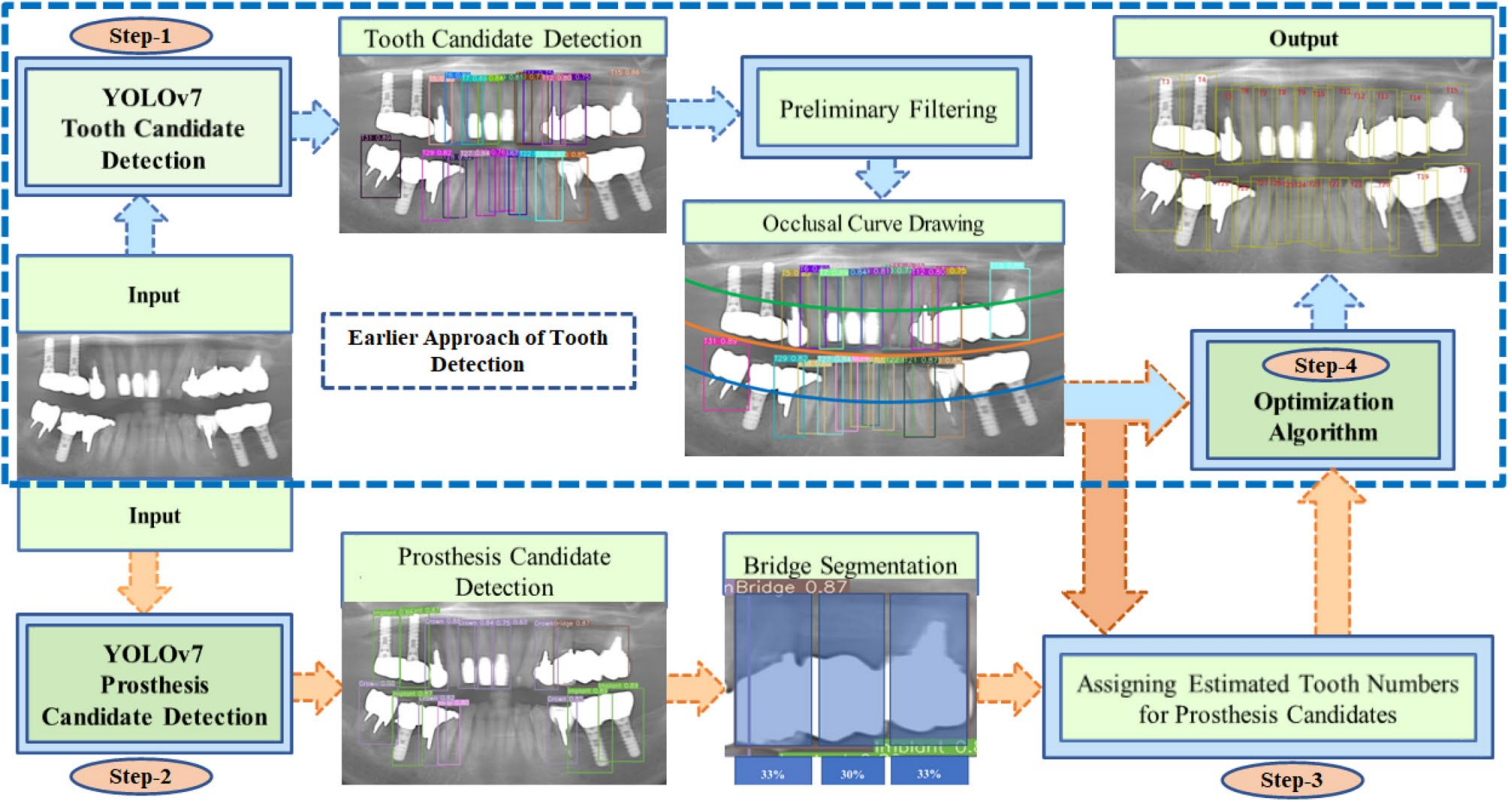
A schematic diagram of the proposed method.

The performance of the traditional teeth detection approach in dental panoramic X-rays is hindered by the alteration of tooth appearance caused by prosthetic treatment^36^. Furthermore, the teeth numbering process^23,25,27^ avoids accounting for complete restorations like implants and fixed dental bridges. Therefore, the actual count of the missing teeth is inappropriate. To address these limitations, this study proposes a novel approach, as depicted in Fig. 4, leveraging two separate CNN-based object detectors known as YOLOv7. One detector is used for detecting tooth candidates, while the other is for detecting prosthesis candidates. A priori knowledge-based algorithm is then employed to optimize the detected candidates, both with and without prostheses. The first approach includes restorations in the recognition process and rechecks of the teeth fitted with prosthetic materials. The latter is the earlier approach to teeth detection^36^. The proposed method involves four steps: (1) detection of tooth candidates using YOLOv7 as a multiple object detection problem in a single dental panoramic image; (2) detection of prosthesis candidates using a separate YOLOv7 network in the same image; (3) assigning approximate tooth numbers to prosthesis candidates based on information obtained from the tooth candidate detector; and (4) combinatorial optimization of the candidates obtained in Steps 1 and 3. Thus, the proposed approach improves the efficiency of teeth detection in dental panoramic X-rays by rechecking teeth that have undergone prosthetic treatment. To investigate the performance improvement, teeth detection is also performed utilizing the earlier approach, where Steps 2 and 3 are ignored. The steps are described below.

### Tooth candidate detection model

A pre-trained convolutional neural network, YOLOv7, detects tooth candidates in panoramic dental X-rays, treating each of the 32 types of teeth as individual objects. The detected candidates are output as bounding boxes with information such as tooth number, x and y coordinates, width, height, and confidence score μ. In the detection process, the same tooth number may be assigned for multiple teeth, or an individual tooth may be recognized with multiple teeth numbers. Assuming that the oral cavity region with teeth occupies about 55% of the *X-axis* in the dental panoramic image, specific false positive candidates are then eliminated. For instance, T10–T24 teeth candidates are considered false positives within 40% of the image width, and T1 to T7 or T26 to T32 are considered false candidates if they lie above 60% of the image width and are subsequently filtered out. In this step, the detected candidates are considered normal teeth. However, the accuracy is expected to be lower for the teeth treated with prosthetic materials.

### Prosthesis candidate detection model

Our teeth detection approach proposes incorporating an extra prosthesis candidate detector to enhance the detection performance of teeth having treatment scares and to detect complete restorations. This decision arises from the fact that when employing a single model for both prostheses and teeth type detection, the number of similar classes increases, posing challenges for recognition. To address this, a separate YOLOv7 model is employed to detect four distinct prosthesis types, such as inlay, crown, implant, and bridge, treating it as a four-class object detection problem. Each detected prosthesis candidate, in this case, provides six pieces of information: prosthesis type, confidence score μ; x, y-coordinate, width, and height of the bounding box. This study focuses solely on bridges designed to replace a single missing tooth, where the detection of bridges includes the supportive teeth and denture portions. Therefore, the obtained bounding box of a bridge is segmented into three sections along the *X*-axis at a ratio of 33:30:33, with the middle section being responsible for the denture.

### Assigning approximate tooth numbers to prosthesis candidates

After detecting prosthesis candidates, the next step involves assigning approximate tooth numbers to these candidates. Initially, it is crucial to determine whether these candidates belong to the upper or lower dentition. This is achieved by using the center points of the bounding boxes for the maxillary teeth T1 to T16 and mandibular teeth T17 to T32 obtained from the tooth candidate detection model. These center points are the basis for approximating the upper and lower dentition as quadratic curves, employing the least-squares method. An additive mean of these curves is computed, yielding a quadratic curve representing the occlusion line, with its apex defined as the center of the dentition. In panoramic images, the dentition center is usually near the image center^41^. However, if the apex of the obtained occlusion line deviates more than 5% from the center of the image width, the center of the image width is then considered the center of the dentition. Subsequently, candidates are categorized into either the maxilla or mandible by comparing their y-coordinate values with the occlusion line. Approximate tooth numbers are then assigned depending on the x-coordinate values of the bounding boxes. For instance, in the maxilla, candidates with x-coordinate values less than the dentition center are assigned tooth numbers 1 to 13, while those greater than the dentition center are assigned tooth numbers 3 to 16. Similarly, in the mandible, candidates with x-coordinates below the dentition center are assigned tooth numbers 32 to 20, and those greater than the center point are assigned tooth numbers 29 to 17. Notably, if a tooth has already been detected in step 1, the confidence score of the prosthesis candidate is reduced by half. In this way, tooth numbers are assigned to the prosthesis candidates, and optimization is performed in the subsequent step.

### Combinatorial optimization with a prior knowledge model

The CNN-based single-shot object detector YOLOv7 can provide outstanding recognition performance. However, there could be numerous false positives, such as double detections for a single tooth, or false negatives arising from the non-detection of teeth with partial restorations such as inlay and crown in step 1. To overcome the miss detection of treated teeth and add the complete restorations, step 2 generates additional candidates. Thus, there could be zero, one, or more candidates for a single tooth. To choose one candidate for each tooth, including the missing tooth, the problem is considered a combinatorial optimization problem. Therefore, a candidate optimization algorithm based on prior knowledge is proposed in this research to eliminate false positives. Both the proposed and the earlier approaches to teeth detection have been used to verify the stability and effectiveness of this modified optimization algorithm^36^.

Let a tooth x be numbered between 1 and 32 and have $N(x)$ candidates such as ${c}_{1}^{x},{c}_{2}^{x},\cdots ,{c}_{N\left(x\right)}^{x}$*.* However, for the missing tooth ${c}^{x}$ is $\phi$, and for the present tooth, ${c}^{x}$ is defined by using the output of YOLOv7 in Eq. (1).

1 $${c}^{x}=\left\{X,Y,W,H,\mu ,R\right\},$$

where *X* and *Y* are the center coordinates of the candidate bounding box, *W* is the width, *H* is the height, $\mu$ is the confidence score, and *R* is the type of prosthesis (normal, inlay, crown, implant, and bridge).

Considering the missing case and sorting the candidates in the descended order based on their confidence score, the candidate combination for tooth $x$ becomes ${c}^{x}\in \left\{\phi ,{c}_{1}^{x},{c}_{2}^{x},\cdots ,{c}_{N\left(x\right)}^{x}\right\}$. Then the candidate combination ${\rm P}$ for 32 teeth is defined by the Eq. (2).

2 $$P=\left\{{c}_{1}^{1},{c}_{1}^{2},\cdots ,{c}_{1}^{32}\right\}.$$

**Figure 5:**
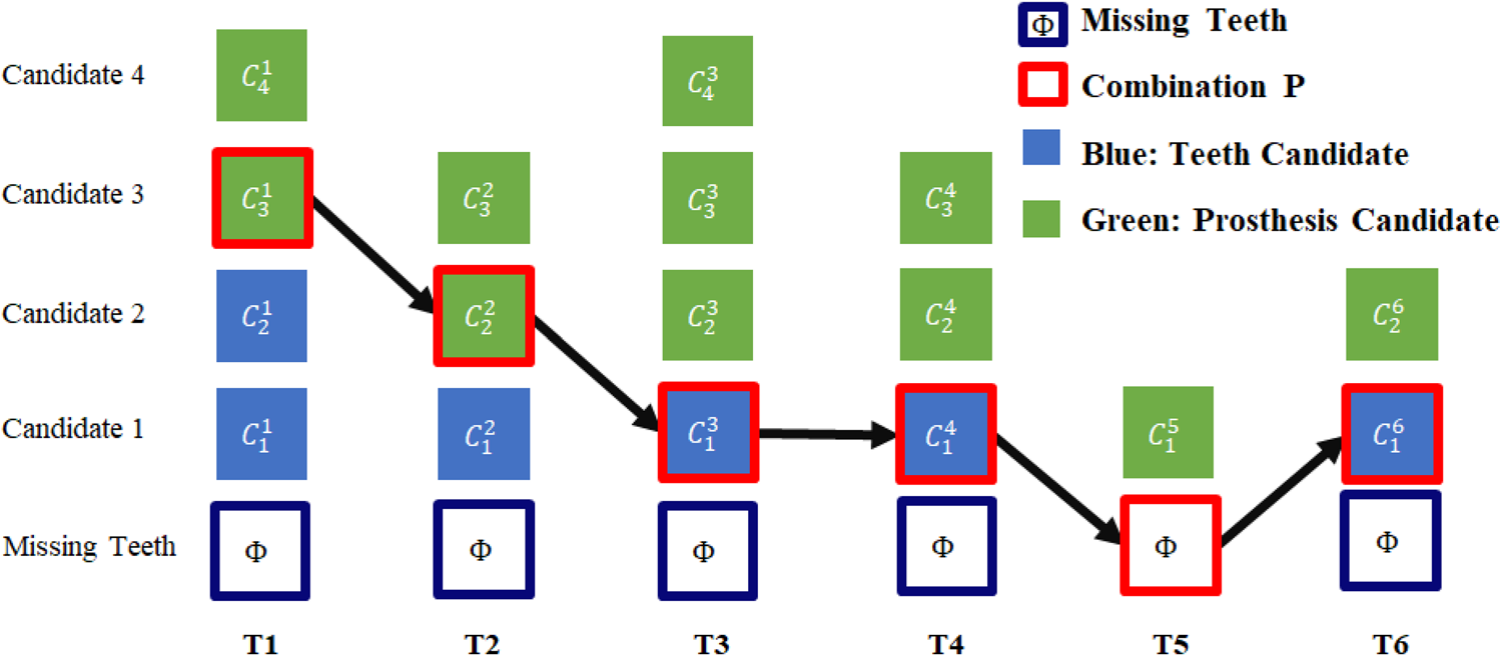
Examples of candidates and combinations before combinatorial optimization (Superscripts denote tooth number).

A simplified representation for the teeth T1 to T6 is given in Fig. 5 to illustrate the relationship between ${c}^{x}$ and the combination ${\rm P}$. The next section delineates the objective function formulated for this research and the implemented optimization algorithm.

#### Objective function based on prior knowledge

We propose an objective function $f\left(P\right)$ in Eq. (3) that evaluates a given combination *P*.

3 $$f\left(P\right)={\omega }_{p}{f}_{p}\left(P\right)+{\omega }_{c}{f}_{c}\left(P\right).$$

Here, the objective function ${f}_{p}\left(P\right)$ is evaluated from the relative position between the teeth candidates, and ${f}_{c}\left(P\right)$ is evaluated from the confidence score of each candidate. ${\omega }_{p}$ and ${\omega }_{c}$ are weight parameters and are determined experimentally^36^.

The following is a description of ${f}_{p}\left(P\right)$. The positional relationship of each tooth's center coordinates is assessed based on prior knowledge. For example, T1 and T2 stand for the tooth-bearing number 1 and 2, respectively. It is expected that T1 is always on the left of T2. In other words, T1 is always located in a position with a smaller x-coordinate than T2. The combination *P* is an unlikely candidate combination if the x-coordinate of the candidate for T1 is greater than the x-coordinate of the candidate for T2. The consecutive candidates that are too far or close to one another are also unsuitable. Therefore, we define an evaluation function ${f}_{p}\left(x\right)$ based on the relative position in Eq. (4) for a tooth number $x$.

4 $${f}_{p}\left(x\right)=\frac{1}{\left|\Omega \right|}\sum_{y\in \Omega }\delta \left(x,y\right).$$

**Figure 6:**
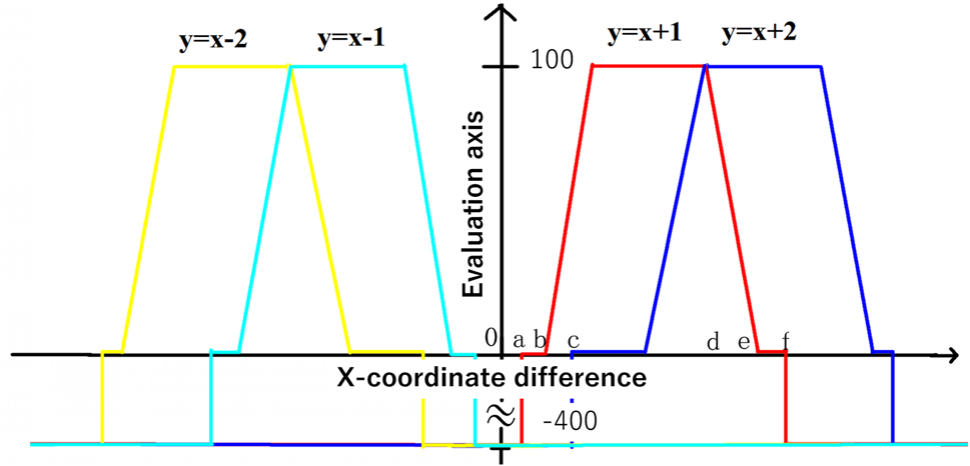
Prior knowledge model for maxilla.

For tooth in the maxilla, $x$ will be ($1\le x\le 16$), and $\Omega =\{x-2,x-1,x+1,x+2\}$. Likewise, for tooth in the mandible, $x$ will be ($17\le x\le 32$), and $\Omega =\{x+2,x+1,x-1,x-2\}$. If neither maxilla nor mandible is applicable, the value is set to 0. The value of $\delta \left(x,y\right)$ is defined in Eq. (5). For the maxilla, it is illustrated in Fig. 6.

5 $$\delta (x,y) = \left\{ {\begin{array}{*{20}l} { - 4,} \hfill & {\left| {P_{x} \left( x \right) - P_{x} \left( y \right)} \right| < a} \hfill \\ {0,} \hfill & {a \le \left| {P_{x} \left( x \right) - P_{x} \left( y \right)} \right| < b} \hfill \\ {\frac{{\left| {P_{x} \left( x \right) - P_{x} \left( y \right)} \right| - b}}{c - b},} \hfill & {b \le \left| {P_{x} \left( x \right) - P_{x} \left( y \right)} \right| < c} \hfill \\ {1,} \hfill & {c \le \left| {P_{x} \left( x \right) - P_{x} \left( y \right)} \right| < d} \hfill \\ {1 - \frac{{\left| {P_{x} \left( x \right) - P_{x} \left( y \right)} \right| - d}}{e - d},} \hfill & {d \le \left| {P_{x} \left( x \right) - P_{x} \left( y \right)} \right| < e} \hfill \\ {0,} \hfill & {e \le \left| {P_{x} \left( x \right) - P_{x} \left( y \right)} \right| < f} \hfill \\ { - 4,} \hfill & {f \le \left| {P_{x} \left( x \right) - P_{x} \left( y \right)} \right|} \hfill \\ {\kappa ,} \hfill & {x = \phi \,\,or\,\,y = \phi } \hfill \\ \end{array} } \right.$$

The experimental values *a, b, c, d, e,* and* f* are adjusted to enhance performance compared to the earlier study^36^. This adjustment is grounded in considering presumed presence of teeth within a specific range. Notably, approximately 55% of the *X-axis* in the dental panoramic image comprises the oral cavity region containing teeth, organized into two rows, each accommodating 16 teeth along the *X-axis* direction. Based on this, *Range* has been defined wherein the presence of teeth is deemed reasonable, signifying the zone where positive results are typically achieved during evaluation. This *Range* is explicitly defined in Eqs. (6), (7) and (8), with *Width* representing the input image’s width.

6 $$b=Width\times 0.55\div 16\times 0.4,$$

7 $$e=Width\times 0.55\div 16\times 2,$$

8 $$Range=e-b.$$

Next, consider the case when the tooth *y* to be compared is very close to tooth *x* or its position is reversed. The tooth $y$ is not selected as the optimal solution even if it has a high score with the other comparison target, and the evaluation score is greatly reduced. The following values are set for such distances.

9 $$a=b/2.$$

The range from the first to the third quartile of $Range$ is defined as the range with the highest evaluation (i.e. the evaluation value of 100) when the tooth is present at the relative position. The range is defined as follows.

10 $$c=b+\left(Range\div 4\right),$$

11 $$d=c+\left(Range\div 4\times 3\right).$$

Finally, if the tooth $y$ to be compared is too far from the tooth $x$, the evaluation points are greatly reduced. Such a distance is evaluated by Eq. (12).

12 $$f=e+\left(Range\div 4\right).$$

When the tooth, $y$ to be compared such that $y=x-2$ or $y=x+2$, the value of $c$ is used instead of $a$, and $c$ is added to each value except $a$. Moreover, two special cases are considered for the mandible. The first case is that the value of $b$ is halved near the center of the mandible, i.e. $21\le x\le 28$, where teeth tend to be crowded. The second case is that the value of $e$ is multiplied by 1.5, when $x$ or $y$ is a mandibular molar, i.e. $17\le x\le 19$ or $30\le x\le 32$. The same calculation is applied to the values other than $b \mathrm{and} e$.

The following is a description of ${f}_{c}\left(P\right)$. YOLOv7 detects an object and identifies the object type if the confidence score exceeds a predetermined threshold. However, for multiple detections of a single object, the result with the highest confidence score is not necessarily correct. Therefore, the confidence score is considered in evaluating the relative coordinates and evaluated comprehensively. ${f}_{c}\left(P\right)$ is defined in Eq. (13) as the average of the confidence scores for 32 teeth in a combination *P*.

13 $${f}_{c}\left(P\right)=\frac{1}{32}{\sum }_{x=1}^{32}\mu \left(x\right).$$

${f}_{c}\left(P\right)$ uses the confidence score obtained by YOLOv7.

#### Combinatorial optimization algorithm

Teeth recognition is performed by finding the combination $P$ that maximizes the evaluation function $f\left(P\right)$. However, there are ${\prod }_{x=1}^{32}{N}_{c}\left(x\right)$ possible combinations which is a large number. Therefore, optimization is performed using the heuristic optimization method as described below.

*Step 0* Initialize a combination ${P=P}_{max}=\{{c}_{1}^{1},{c}_{1}^{2},{c}_{1}^{3},\dots ,{c}_{1}^{32}\}$ where the superscript of *C* is the tooth number, and the subscripts of *C* are the candidates with the height confidence score. If there is no candidate, the corresponding tooth is assumed to be missing and denoted by $\phi$. The objective function $f\left(P\right)$ for the combination *P* is calculated using Eq. (3).

*Step 1* Let tooth number *x* be 1.

*Step 2* For ${c}^{x}\in \left\{\phi ,{c}_{1}^{x},{c}_{2}^{x},\cdots ,{c}_{N\left(x\right)}^{x}\right\}$, let $P$′ be the combination in which the tooth $x$ in combination $P$ is replaced by ${c}^{x}$, and calculate the evaluation value $f{\prime}$. Here, let the combination with the highest evaluation value is ${f(P)}_{max}$ for the combination ${P}_{max}$.

*Step 3* When $f{\prime}>{f(P)}_{max}$, the combination $P\boldsymbol{^{\prime}}$ is updated with ${P}_{max}$ and $f{\prime} \mathrm{with} {f(P)}_{max}$.

*Step 4* Add 1 to $x$, and return to Step 2 until $x\le 32$.

*Step 5* Repeat Step 2 through Step 4 N times or until there are no more updates.

*Step 6* If the combination ${P}_{max}$ contains a missing tooth and there are other options, perform a round-robin search for the target tooth and its four neighboring teeth.

*Step 7* If ${P}_{max}$ shows no teeth on either side of tooth x, and only one tooth exists on both sides, an exception is made. The tooth is selected based on its proximity in number to the tooth that is closer in distance to tooth x.

### Evaluation metrics

Precision (*P*), recall (*R*), intersection over union (*IOU*), average precision (*AP*), and mean average precision (*mAP*) are frequently employed metrics within the field of object detection. The *IOU* measures the similarity of detected and ground truth bounding boxes. When the value of *IOU* is one, it indicates a perfect match; when the value is zero, it implies no match. In Eq. (14), the *IOU* is defined as the ratio of the intersection and union areas between the ground-truth bounding box ${B}_{g}$ and the predicted bounding box ${B}_{p}$.

14 $$IOU=Area\,\, of\,\, ({B}_{p}\bigcap {B}_{g})/Area \,\,of\,\, ({B}_{p} U {B}_{g})$$

However, the assessment of object detection methods is mainly based on the precision *P* and recall *R*, defined in Eqs. (15) and (16), respectively.

15 $$P=\frac{TP}{TP+FP}=\frac{TP}{all\,\, detection},$$

16 $$R=\frac{TP}{TP+FN}=\frac{TP}{all\,\, ground \,\,truths},$$

In this research, the detected box was considered as true positive (*TP*) if it had an *IOU* of 0.5 or greater with proper class, false positive (*FP*) for an incorrect class or misplaced detection, and false negative (*FN*) if it was undetected or had an *IOU* less than 0.5.

The *AP* metric summarizes the trade-off between precision and recall based on the confidence scores of predicted boxes. All-point interpolation was used to compute *AP* by interpolating through all points in such a way that:

17 $${AP}_{all-point\,\, interp}=\sum_{n}({R}_{n+1}-{R}_{n}){P}_{interp}{(R}_{n+1}),$$

where $${P}_{interp}{(R}_{n+1})=\frac{max}{{R}^{\mathrm{^{\prime}}}:{R}^{\mathrm{^{\prime}}}\ge {R}_{n+1}}P({R}^{\mathrm{^{\prime}}})$$

To represent the exactness of the detections across all classes, the mean average precision (*q*) was computed, which is simply the sum average of *AP* over all categories, that is,

18 $$mAP=\frac{1}{N}\sum_{i}^{N}{AP}_{i}.$$

The harmonic mean of the precision and recall is termed the F-1 score and is defined by the Eq. (19). To assess the strength and consistency of the candidate optimization algorithm and to compare the performance enhancement between the proposed and earlier teeth detection approaches, the F-1 score was utilized.

19 $${F}_{1}Score=\frac{2\times (P\times R)}{(P+R)}.$$

All these metrics had been calculated using the open-source tool-kit^42^ which was used as the official tool in city intelligence hackathon competition and part of the algorithm was implemented in YOLOv5^43^.

### Ethics declarations

This study was conducted with the approval of the Institutional Review Board (IRB) at Kashiwanohara Sogo Dental in Chiba, Japan, and the IRB at Narcorm Co. Ltd (Approval number: N20180319-HG0001). Informed consent was waived by the IRB at Kashiwanohara Sogo Dental in Chiba, Japan. The images were collected anonymously by Narcorm Co. Ltd, ensuring that no additional information such as age, gender, or height was disclosed. Moreover, this research adhered to all relevant laws, regulations, and rules.

## Results

This section presents the experimental environment, training parameter settings, and extracted outcomes.

### Experimental environment

In this paper, all experiments were conducted on a computer running a 64-bit Windows 10 operating system. The CPU was configured with an AMD Ryzen-7 2700 Eight-Core Processor with a clock speed of 3.20 GHz, 32 GB RAM, and an NVIDIA GeForce RTX 2080 Ti graphics card with 11 GB of video memory. The software environment comprised Python 3.9.13, PyTorch 1.12.1, and CUDA 11.3.1.

### Training parameter settings

All the training processes adopted pre-trained weights on the COCO dataset. The entire YOLOv7 architecture was trained since the dataset was large enough and differed significantly from the COCO dataset. The training processes leveraged a stochastic gradient descent optimizer with a default momentum of 0.937 and weight decay of 0.0005 to update the convolution kernel parameters. The initial and final one-cycle learning rates were 0.01 and 0.1, respectively. The box loss, objectness loss, classification loss, and *IOU* training threshold values were 0.05, 0.7, 0.3, and 0.2, respectively. The network received images with a resolution of 640 × 640 pixels. This paper selected a batch size of 12 to maximize the amount of data based on the GPU configuration. To reduce the overfitting effect, we mainly used Mosaic, Translate, Scale, and Mixup augmentation techniques from the default image augmentation settings of YOLOv7.

### Experimental results

For a clearer understanding, the extracted results of our research have been presented in two separate sections. In all test experiments, the YOLOv7 inference algorithm was utilized with a confidence score of 0.35 for object detection.

#### First experimental results

In the first experiment, we evaluated the tooth and prosthesis candidate detection models built on the CNN-based object detector YOLOv7. Train set A, consisting of 2615 panoramic radiographs, was used to train both networks separately utilizing the early stopping technique by setting epoch-patience value 30. The best weights were achieved at 39 and 25 epochs for tooth and prosthesis candidate detection models, respectively. Both the tooth and prosthesis candidate detection networks underwent testing using test set A. Additionally, the tooth candidate detection model was evaluated using test set E to confirm the model's performance on data obtained from an external source.

**Figure 7:**
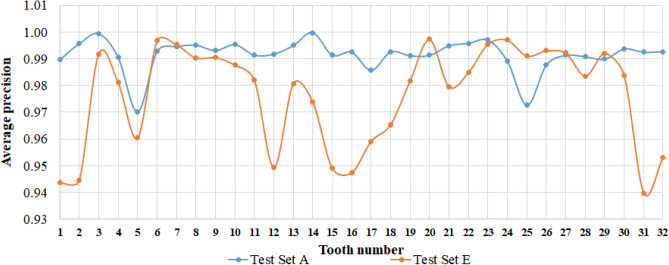
Average precision for 32 teeth categories plotted using test datasets A and E.

The *mAP* of four different prosthesis categories was 97.36%, with a 100% precision value for the bridge detection. Although implant detection was 100%, five false positives yielded a low precision value. Table 1 presents precision and recall values for each category and the number of true positives, false positives, and average precisions in test set A. The *mAP* of the tooth candidate detection model for test sets A and E were 99.15% and 97.67%, respectively. Table 2 presents the precision and recall values for each of the 32 teeth categories for test sets A and E. Figure 7, plots *APs* of 32 teeth categories for both test datasets A and E.

**Table 1:** Performance evaluation of prosthesis candidate detection model for test set A.

Metrics	Inlay	Crown	Implant	Bridge
Number of instances	2349	864	59	86
TP	2264	846	59	85
FP	117	70	5	0
Precision	0.95	0.92	0.92	1.00
Recall	0.96	0.98	1.00	0.99
Average precision	0.96	0.96	0.99	0.99

**Table 2:** Performance evaluation of tooth candidate detection model for test sets A and E.

Tooth number	Test set A		Test set E		Tooth number	Test set A		Test set E	
	Precision	Recall	Precision	Recall		Precision	Recall	Precision	Recall
T1	0.97	0.99	0.95	0.95	T17	0.95	0.99	0.94	0.96
T2	0.99	1.00	0.89	0.97	T18	0.99	1.00	0.92	0.97
T3	0.99	1.00	0.94	1.00	T19	1.00	0.99	0.93	1.00
T4	0.97	1.00	0.92	0.99	T20	0.98	0.99	0.98	1.00
T5	0.96	0.97	0.9	0.96	T21	0.98	1.00	0.95	0.98
T6	0.98	1.00	0.95	1.00	T22	0.98	1.00	0.96	1.00
T7	0.97	1.00	0.94	1.00	T23	0.96	1.00	0.97	1.00
T8	0.98	1.00	0.98	0.99	T24	0.95	0.99	0.96	1.00
T9	0.99	1.00	0.97	0.99	T25	0.94	0.98	0.97	0.99
T10	0.98	1.00	0.96	0.99	T26	0.94	0.99	0.97	0.99
T11	0.98	0.99	0.95	0.98	T27	0.96	0.99	0.96	1.00
T12	0.97	0.99	0.91	0.95	T28	0.98	0.99	0.95	0.99
T13	0.97	1.00	0.9	0.99	T29	0.98	0.99	0.98	0.99
T14	0.99	1.00	0.95	0.98	T30	1.00	1.00	0.93	1.00
T15	0.98	0.99	0.92	0.96	T31	0.98	0.99	0.91	0.95
T16	0.97	1.00	0.94	0.96	T32	0.98	1.00	0.95	0.96

#### Second experimental results

In the second experiment, we utilized a six-fold cross-validation (CV) to test the compatibility and robustness of the candidate optimization algorithm in two different approaches and assess the feasibility of the proposed method. The first approach involved tooth and prosthesis candidate detection using two individual YOLOv7 detectors, followed by an optimization technique to improve the overall recognition results, referred to as the proposed method. The second approach involved teeth recognition through the YOLOv7 tooth candidate detector, followed by the optimization algorithm, an approach we had previously employed in our prior teeth detection research^36^, and hereafter referred to as the earlier approach. In each training period of six-fold CV, the tooth and prosthesis candidate detection models were trained separately with epoch numbers 39 and 25, respectively. The candidate optimization algorithm^36^ based on prior knowledge was modified and implemented to refine the candidates obtained by YOLOv7 models, with ${\omega }_{p}$ and $\omega_{c}$ values set at 0.8 and 0.2, respectively. The performance of both approaches (columns 2 and 4) was compared with the outcome of the tooth candidate detection model (column 5), as presented in Table 3.

**Table 3:** Six-fold cross-validation results.

Ground truth			All annotation			Tooth annotation			Tooth annotation			Tooth annotation		
Test set	Number of instances		Proposed method			Proposed approach (Without complete restorations)			Earlier approach			Tooth candidate detection model		
	All Annotation	Tooth Annotation	P	R	F1 score	P	R	F1 score	P	R	F1 score	P	R	F1 score
Fold 1	15,132	14,975	0.9836	0.9854	0.9845	0.9860	0.9877	0.9868	0.9841	0.9852	0.9847	0.9639	0.9925	0.9780
Fold 2	15,141	14,962	0.9854	0.9877	0.9866	0.9863	0.9892	0.9878	0.9841	0.9862	0.9851	0.9658	0.9936	0.9795
Fold 3	15,140	14,988	0.9850	0.9886	0.9868	0.9865	0.9905	0.9885	0.9840	0.9870	0.9855	0.9638	0.9941	0.9788
Fold 4	15,109	14,988	0.9846	0.9872	0.9859	0.9860	0.9887	0.9874	0.9842	0.9869	0.9856	0.9652	0.9939	0.9793
Fold 5	15,122	14,972	0.9851	0.9892	0.9871	0.9865	0.9906	0.9885	0.9846	0.9881	0.9864	0.9618	0.9948	0.9780
Fold 6	15,012	14,838	0.9817	0.9845	0.9831	0.9835	0.9862	0.9848	0.9806	0.9823	0.9815	0.9568	0.9921	0.9741
Average			0.9842	0.9871	0.9857	0.9858	0.9888	0.9873	0.9885	0.9860	0.9848	0.9629	0.9935	0.9780

**Figure 8:**
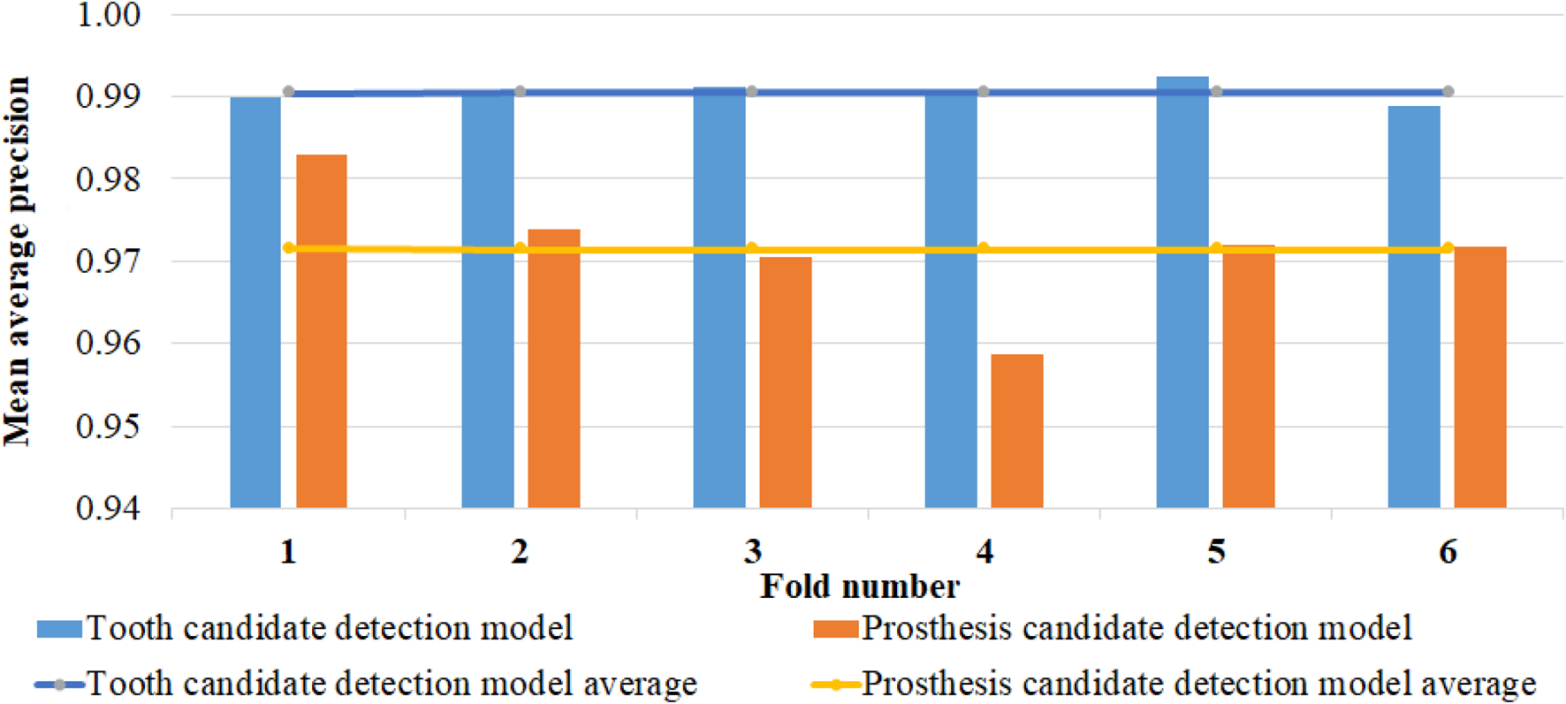
Mean average precision of six different folds for tooth and prosthesis candidate detection model.

The obtained results, including the *AP* of all teeth categories and prosthesis types in six different test folds for tooth and prosthesis candidate detection models, are summarized in Fig. 8. The average *mAPs* for the tooth and prosthesis detection model were 99.06% and 97.16%, respectively.

**Figure 9:**
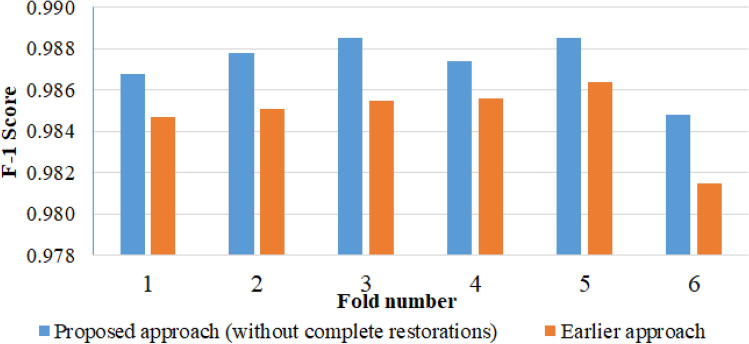
Comparison between the proposed and earlier approach of tooth detection in terms of F1-score.

An additional analysis was conducted to explore the performance improvement of the proposed teeth detection approach compared to the earlier approach. In this analysis, we excluded the complete restorations (implant and denture) from the detected outcomes of the proposed method in six different test folds. Then, we evaluated the performance with respect to the Tooth Annotation ground truths for each test fold. The comparative results are presented in columns 3 and 4 of Table 3 and illustrated in Fig. 9 using the F1-score.

## Discussion

**Figure 10:**
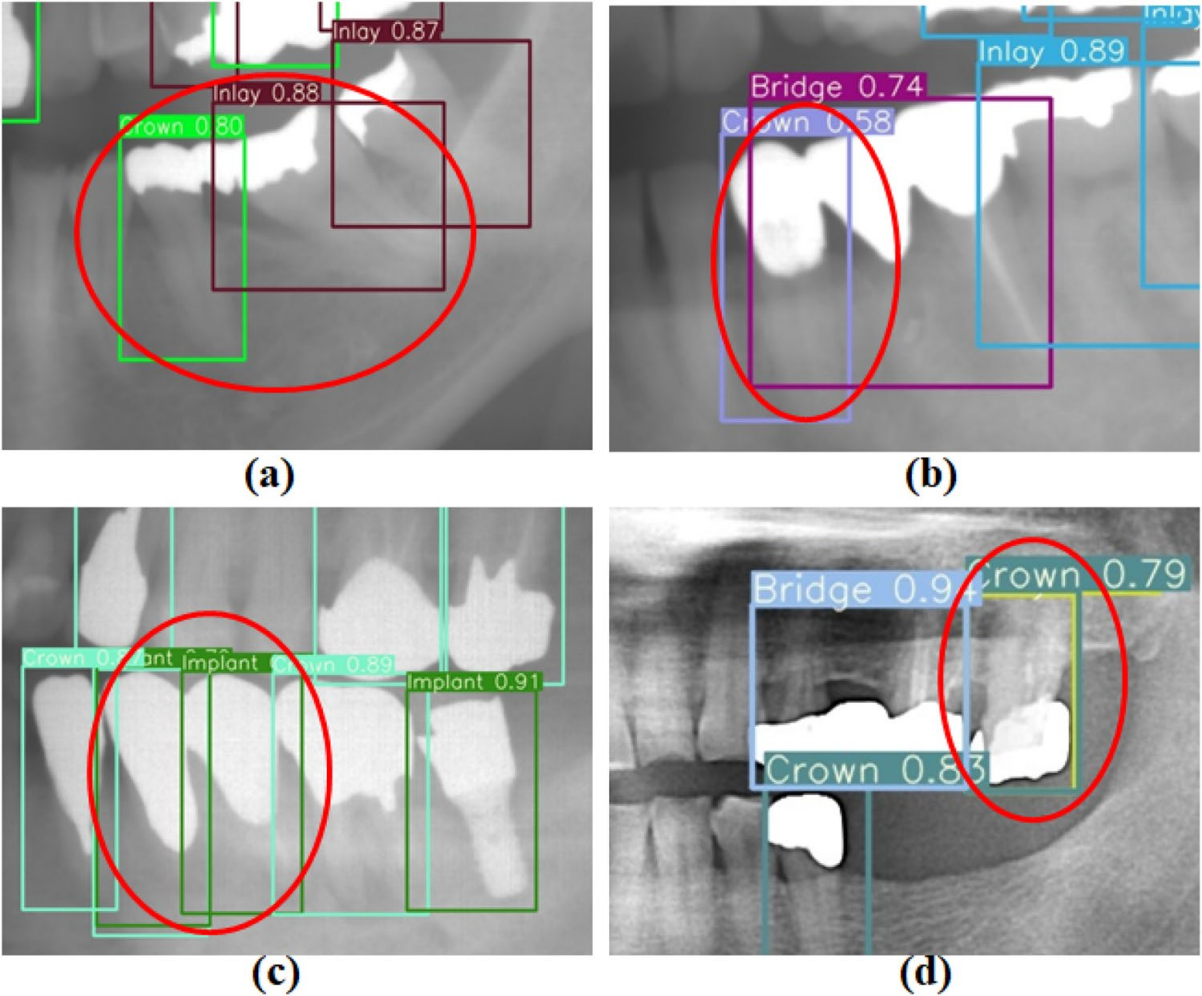
Examples of challenging cases encountered in the prosthesis candidate detection model.

The research presented in this paper aimed to develop an automated system for teeth, including prosthesis detection and enumeration, using two distinct YOLOv7 models with a robust optimization algorithm. The study utilized panoramic radiographs and verified using an external dataset E. Table 1 indicates high recall values but relatively low precision values for implants and crowns in the prosthesis candidate detection model. This is because crowns were incorrectly detected as implants and cross-classified with inlays, especially with onlays/partial crowns. Figure 10 illustrates some limitations of the prosthesis detection model: (a) shows an undetected bridge, (b) demonstrates an inlay falsely detected as a crown, (c) shows a crown falsely identified as an implant, and (d) shows a bridge basement identified as a crown. However, despite these limitations, the high *mAP* value indicates that the model could effectively detect prosthesis candidates.

**Figure 11:**
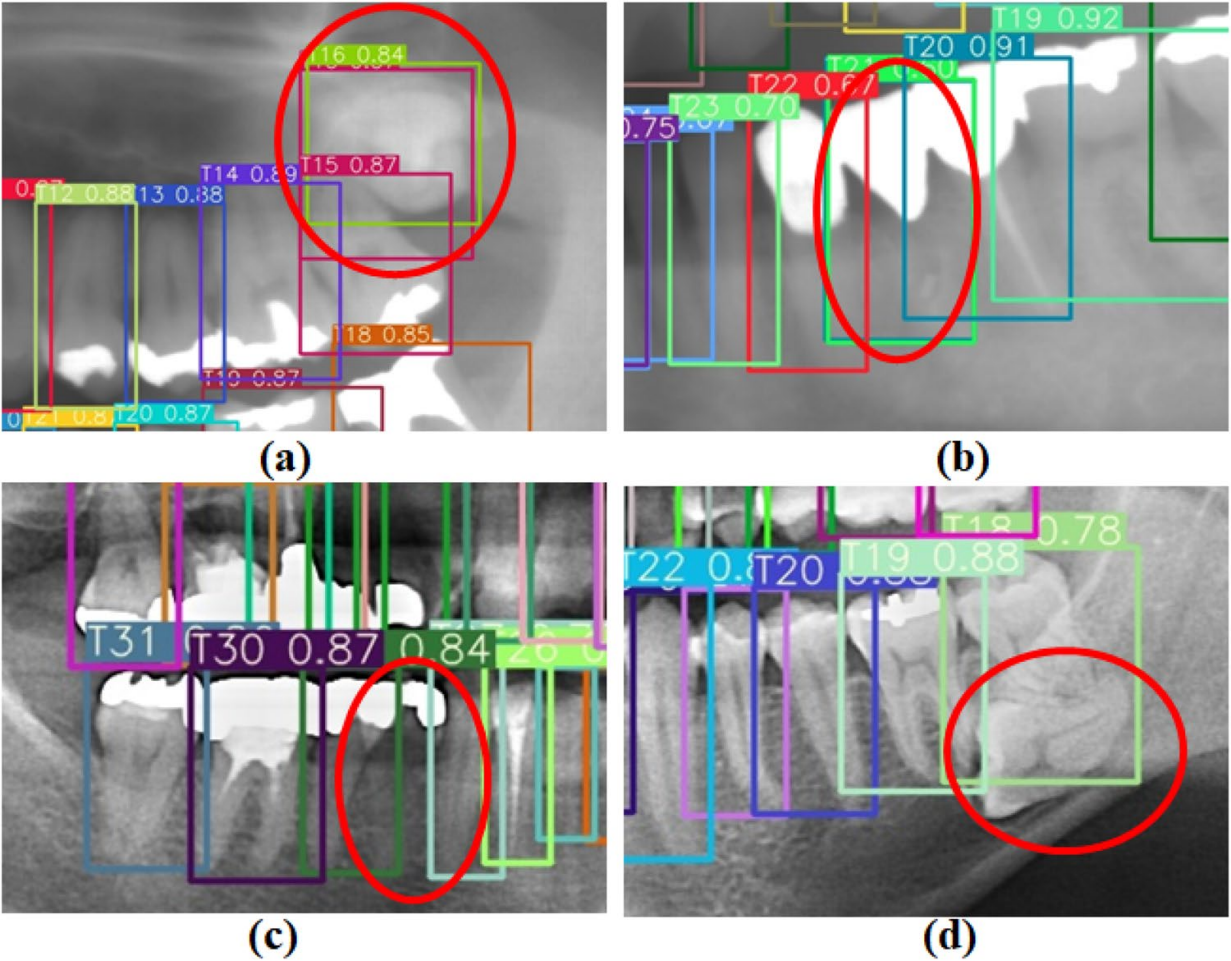
Examples of challenging cases encountered in the tooth candidate detection model.

The tooth candidate detection model exhibited strong performance on test set A, achieving perfect precision for T3 and T14, as well as high recall for most teeth as provided in Table 2. However, when applied to test set E, the model encountered issues, specifically regarding missing molars, which led to incorrect numbering of adjacent teeth and resulted in low performance, as depicted in Fig. 7. Furthermore, the average number of teeth per image was 26.59 in test set E, compared to 28.59 in test set A, contributing to the low performance on test set E. The premolar teeth, which have similar shapes, were prone to be incorrectly numbered due to their proximity. Some labels in the train and test data were contentious, especially when a premolar tooth was missing without leaving space, making it difficult to establish correct ground truth labels. In test set A, the overlapping of T25 also contributed to low performance. Additionally, the tooth candidate detection model encountered several challenges, including multiple detections of a single tooth, false detection of bridge dentures, difficulty detecting prosthetic-treated teeth, and failure to detect misaligned teeth, as evidenced in Fig. 11a–d.

**Figure 12:**
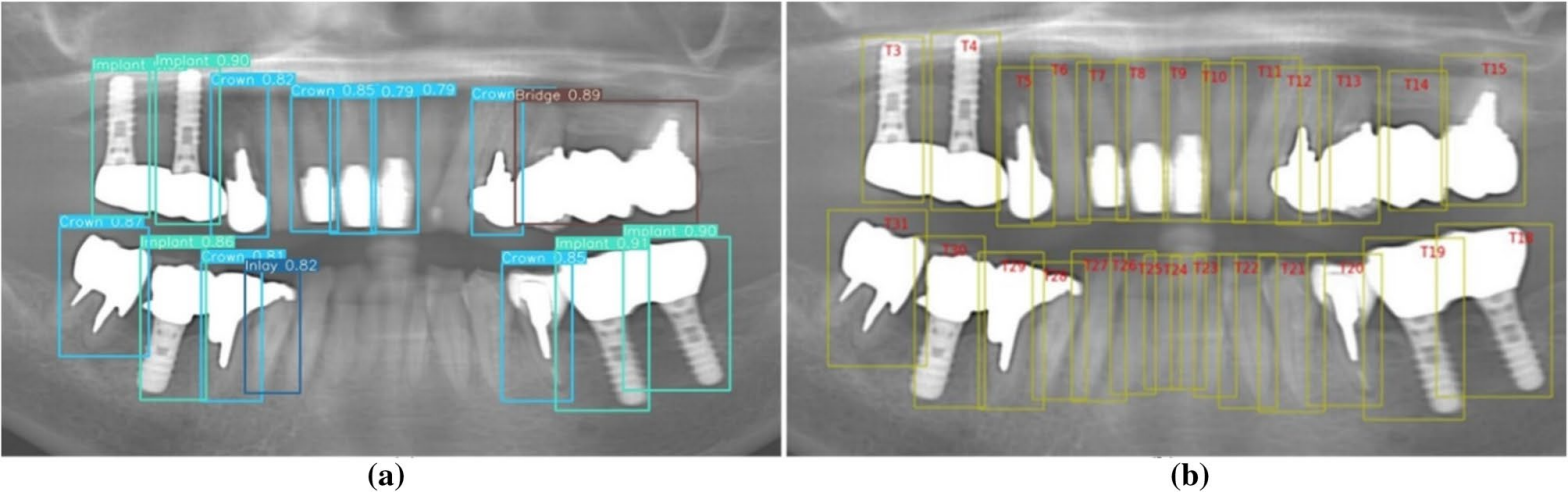
Illustration of successful (**a**) prosthesis detection and (**b**) proposed method outcome.

Table 3 presents the six-fold CV results, demonstrating that the optimization algorithm improved the stability and balance of the proposed and earlier teeth detection approaches compared to relying solely on deep learning-based detection. The tooth candidate detection model exhibited exceptional performance in the six-fold cross-validation, with no deviation from an average *mAP* of 99.06%, as illustrated in Fig. 9. However, the prosthesis detection model in Fold-4 exhibited a minor negative deviation from the average *mAP* of 97.16%. This deviation could be attributed to porcelain prostheses instances in that fold, which were few in the dataset and went undetected. Conversely, Fold-1 exhibited a higher performance than the average, likely because the cross-classification cases between the inlay and crown were relatively low. A comparison between the proposed and earlier teeth detection approaches is shown in Table 3 and Fig. 9. Despite teeth deformation caused by prosthetic treatment, with a significant number of treated teeth (196,021 instances in 2553 images), the tooth candidate detection model was effectively trained, resulting in insignificant performance improvement. However, the proposed model detected and enumerated complete restorations (implants and dentures), leading to a more accurate count of missing teeth than other studies. Overall, the proposed method is novel in its approach to teeth and prosthesis detection, as well as in its consideration of restorations in the teeth enumeration process according to the UTN system. Figure 12 illustrates the successful outcome of the proposed method.

## Conclusion

In conclusion, the proposed method is unique in detecting and enumerating teeth, including complete dental restorations. This method leverages two distinct CNN-based object detectors, namely YOLOv7, for precisely detecting teeth and prostheses. Additionally, it integrates a candidate optimization algorithm based on prior knowledge, resulting in outstanding *mAP*s of up to 0.992 for teeth detection and 0.983 for prosthesis detection. Through the application of six-fold cross-validation, both with and without the candidate optimization technique, this study demonstrates the remarkable effectiveness of the implemented optimization algorithm in enhancing overall detection performance. Moreover, the investigation illustrates that integrating prosthesis information into the teeth detection process improves detection performance. The inclusion of prosthesis data allows the proposed teeth detection approach to enumerate complete restorations and accurately determine the count of missing teeth, positioning the method as a prospective tool for automating dental chart creation^44^. Nevertheless, it is crucial to acknowledge certain limitations of this research. For instance, the method requires dental X-rays containing at least five teeth in both the upper and lower jaws to accurately trace the occlusal curve for accommodating the prostheses properly. Additionally, challenges remain in segmenting bridge sections with more than two dentures. Broken or residual roots should also be considered in the enumeration process. In the future, our plan encompasses overcoming these challenges and expanding the scope of prosthetic features to develop a comprehensive dental chart that can further advance dental diagnostic capabilities.

## Data Availability

The findings of this study were supported by data that was obtained from Narcorm Co. Ltd., but there are certain restrictions on its availability due to its use under the agreements for the current study. Therefore, the data is not publicly accessible. However, upon reasonable request and with the approval of Narcorm Co. Ltd., the corresponding author can provide access to the data.
